# Acupuncture Modulation of the Lung–Brain Axis in Alzheimer’s Disease: Mechanisms and Therapeutic Perspectives

**DOI:** 10.3390/brainsci15101076

**Published:** 2025-10-03

**Authors:** Jiaxing Zou, Ruiwei Liao, Wen Zhang, Zaoyuan Kuang

**Affiliations:** School of Basic Medical Sciences, Guangzhou University of Chinese Medicine, No. 232, East Waihuan Road, Guangzhou Higher Education Mega Centre, Panyu District, Guangzhou 510006, China; zoujiaxing@stu.gzucm.edu.cn (J.Z.); liaoruiwei@stu.gzucm.edu.cn (R.L.); zhangw@gzucm.edu.cn (W.Z.)

**Keywords:** acupuncture, lung-brain axis, Alzheimer’s disease, Chinese medicine, neuroinflammation, cognitive impairment

## Abstract

Alzheimer’s disease (AD) is a prevalent neurodegenerative disorder characterized by progressive cognitive decline and an impaired quality of life, for which no curative treatment is currently available. Recent research indicates that chronic pulmonary conditions—including chronic obstructive pulmonary disease (COPD) and obstructive sleep apnea (OSA)—exhibit significant epidemiological associations with AD pathogenesis, suggesting that the lung–brain axis may contribute to AD development. Acupuncture, a core TCM intervention, shows promise for modulating multisystem functions and enhancing cognitive performance. This review synthesizes the current evidence regarding pulmonary diseases influencing AD through the lung–brain axis, elucidates potential mechanisms by which acupuncture may modulate pulmonary function and mitigate AD pathology, and explores future directions for lung–brain axis-targeted acupuncture interventions. Our overarching aim is to propose integrative, evidence-based strategies that combine Chinese and Western medicine for the prevention and management of AD.

## 1. Introduction

As global population aging accelerates, Alzheimer’s disease (AD)—one of the most prevalent neurodegenerative dementias—poses serious threats to elderly quality of life [1,2]. Notably, Alzheimer’s disease prevalence reaches approximately 5% of individuals aged 65+ and rises to 20% among those over 80. Consequently, AD cases are predicted to double every 20 years, reaching an estimated 131.5 million by 2050, which will impose substantial pressure on public health systems [3,4,5,6].

At present, therapeutic options for AD are limited, primarily relying on cholinesterase inhibitors (e.g., donepezil), NMDA receptor antagonists (e.g., memantine), and certain Aβ-clearing antibodies (e.g., aducanumab) [7,8]. However, their clinical efficacy is typically lacking. The majority of these currently available agents exhibit efficacy predominantly in mild-to-moderate AD and fail to halt the underlying neurodegenerative process [9]. Additionally, they are associated with side effects; for example, cholinergic inhibitors may induce adverse reactions such as gastrointestinal spasms or cardiac arrhythmias [10,11,12]. Moreover, although the U.S. Food and Drug Administration (FDA) has approved several anti-Aβ monoclonal antibodies for early intervention in AD, their efficacy in slowing disease progression remains a subject of considerable controversy [13]. Consequently, there is an urgent need to develop innovative intervention strategies characterized by multi-target mechanisms of action.

Recent reappraisals of AD pathogenesis have highlighted that, beyond traditional mechanisms such as genetic susceptibility (e.g., ApoEε4 allele), Aβ deposition, aberrant tau phosphorylation, and central cholinergic system impairment, the “peripheral–central axis” has garnered growing recognition [14,15]. Particularly, the impact of pulmonary dysfunction on cognitive decline has attracted extensive attention [16].

The “lung–brain axis,” a recently developed theory, describes how pulmonary disorders can affect brain function and induce or exacerbate cognitive impairment through multiple pathways, such as chronic hypoxia, the release of inflammatory cytokines, oxidative stress, and the disruption of the blood–brain barrier (BBB) [17,18]. Xia Chen et al. found that the progression of cognitive impairment is significantly accelerated in patients with COPD, and their susceptibility to AD is significantly increased [19]. These converging lines of evidence strongly suggest a direct pathophysiological link between chronic pulmonary pathology and Alzheimer’s neurodegeneration. The underlying mechanisms encompass hypoxia-induced reductions in acetylcholine synthesis, accelerated Aβ deposition, heightened tau protein phosphorylation, and augmented neuronal apoptosis [20]. Furthermore, a declining pulmonary function can elicit systemic oxidative stress, thereby increasing the elevation of superoxide production. This, in turn, exacerbates blood–brain barrier (BBB) disruptions, precipitating to central inflammation and neuronal injury. As highlighted in the seminal work by Sang Ryong Kim et al., oxidative stress represents a hallmark pathological feature in neurodegenerative brains. The accumulation of reactive oxygen species (ROS) overwhelms endogenous antioxidant defenses, thereby compromising neuronal membrane integrity and promoting toxic aggregate deposition. Concurrently, oxidative insult induces blood–brain barrier (BBB) dysfunction, substantially enhancing the parenchymal penetration of neurotoxins [21]. Rao Muralikrishna Adibhatla et al. also discussed the issue of oxidative stress and CNS damage as important factors leading to AD. It is well known that patients with COPD, OSA, etc., have high levels of oxidative stress, which provides strong evidence for the “lung–brain axis” [22].

The theory that there is a “lung–brain axis” in Chinese medicine is equally well founded. In Traditional Chinese Medicine (TCM) theory, with ideas like “the lungs govern the vessels” (“fei chao bai mai”), “the brain houses the original spirit” (“nao wei yuan shen zhi fu”), “the lungs are the mother of the kidneys” (“fei wei shen zhi mu”), and “the kidneys govern bones and generate marrow, while the brain is the sea of marrow” (“shen zhu gu sheng sui, nao wei sui hai”), offers theoretical underpinnings for the lung–brain connection. According to earlier studies, declining kidney function also accelerates the course of AD by affecting neurodevelopment and myelination processes [23]. Additionally, impaired pulmonary function, functioning as an “upstream” factor, may serve as a crucial potential trigger for AD through the interconnected “lung–brain–kidney” pathway.

In light of this, Traditional Chinese Medicine (TCM) has shown considerable potential in the treatment of AD. As reported by Zhi-Yong Wang et al., ginsenoside Rg1—an extract derived from ginseng root—reduces Aβ aggregation through the inhibition of γ-secretase activity and also suppresses neuronal apoptosis. Sandeep Kumar Singh et al. noted that extracts from Ginkgo biloba (Gb) exhibit mechanisms such as enhancing neuroplasticity and modulating neurotransmitters, providing an alternative strategy for managing neurodegenerative diseases. These extracts have demonstrated significant therapeutic efficacy in experimental models, with minimal adverse effects. Thus, TCM holds promising value for AD treatment [24,25]. As an important complementary approach within TCM, acupuncture also exhibits unique therapeutic benefits against AD [26]. Contemporary research indicates that acupuncture interventions targeting the Lung Meridian and Back-Shu points (e.g., Feishu BL13, Dingchuan EX-B1, Taiyuan LU9) effectively ameliorate pulmonary function, regulate neuroendocrine and inflammatory cytokine release, and enhance vagal nerve activity alongside HPA axis stability [27,28]. This cascade effect facilitates the positive modulation of the lung–brain axis, which may provide new therapeutic approaches for the treatment of AD.

**Figure 1:**
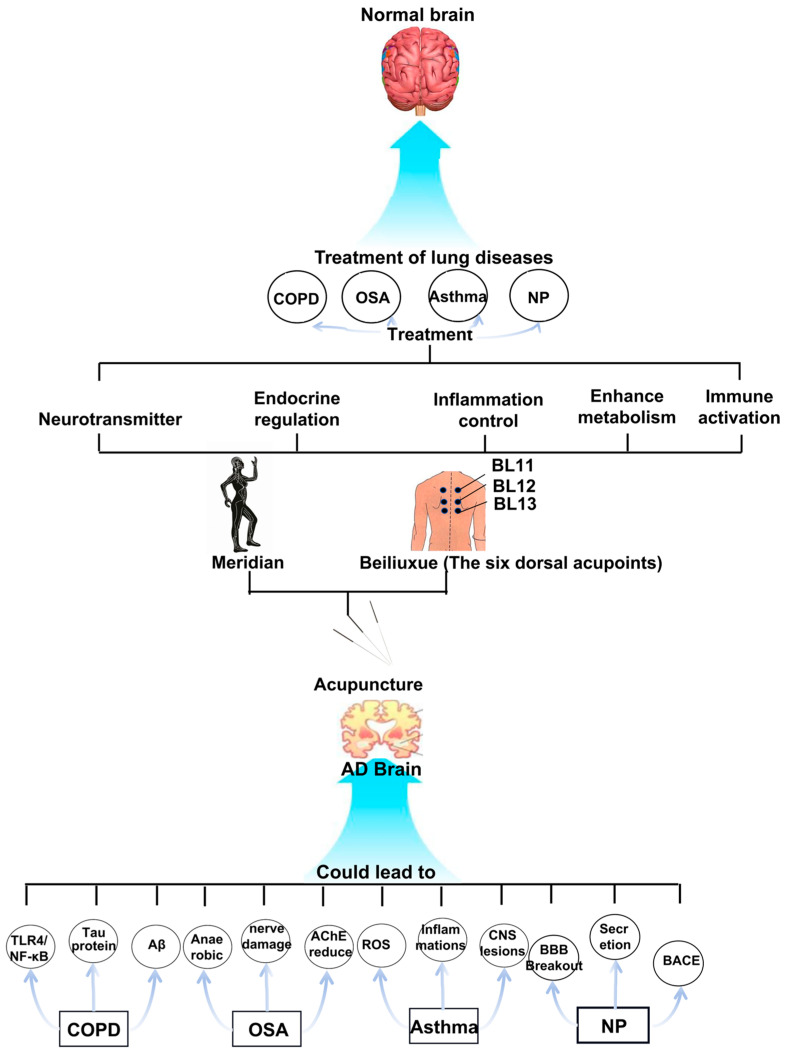
Graphical abstract illustrating the core concept of this review. This schematic illustrates the integrated mechanisms of the lung–brain axis in the pathogenesis of AD. Common pulmonary diseases contribute to or exacerbate AD through several representative mechanistic pathways. Acupuncture, targeting specific meridians and acupoints (e.g., the six dorsal acupoints), may ameliorate pulmonary conditions by potentially promoting neurorestoration, modulating endocrine responses, resolving inflammation, and regulating immune activity. This multi-targeted intervention may subsequently contribute to the restoration of normal brain function in AD. This framework proposes potential strategic directions for future research on acupuncture targeting the lung–brain axis.

As a result, this article summarizes recent findings at the nexus of modern medicine and Traditional Chinese Medicine (TCM) theory. It thoroughly examines the following topics: (1) potential links between pulmonary conditions and AD [29]; (2) the pathophysiology of the lung–brain axis; (3) developments in acupuncture-mediated pulmonary enhancement for AD prevention [30]. This synthesis seeks to establish a theoretical groundwork for novel, multidisciplinary approaches to comprehensive AD prevention and management, providing theoretical underpinnings and conceptual insights (Figure 1).

## 2. Lung–Brain Axis in AD Pathogenesis

### 2.1. The Impact of Chronic Obstructive Pulmonary Disease (COPD) on Alzheimer’s Disease (AD)

Chronic obstructive pulmonary disease (COPD) is a respiratory disorder characterized by a continuous restriction of airflow, often accompanied by chronic hypoxemia and systemic inflammation [31]. Mounting evidence has revealed a significantly higher incidence of cognitive impairment among COPD patients, suggesting a potential link between this disorder and AD [32]. A clinical study by Jing Li, Guang-He Fei et al. carried out thorough evaluations of 85 participants, including pulmonary function tests, arterial blood gas analysis, Montreal Cognitive Assessment (MoCA), serum S100B measurement, and hippocampus MRI [33]. The findings demonstrated hippocampal volume reduction and cognitive impairment in COPD patients. These changes may be closely linked with hypoxia-induced neuronal apoptosis and the diminished expression of neurotrophic factors. The hippocampus—a key brain region implicated in early AD pathology—exhibits a high vulnerability to hypoxia. Its structural damage aligns closely with well-established pathogenic mechanisms of AD [34,35].

**Figure 2:**
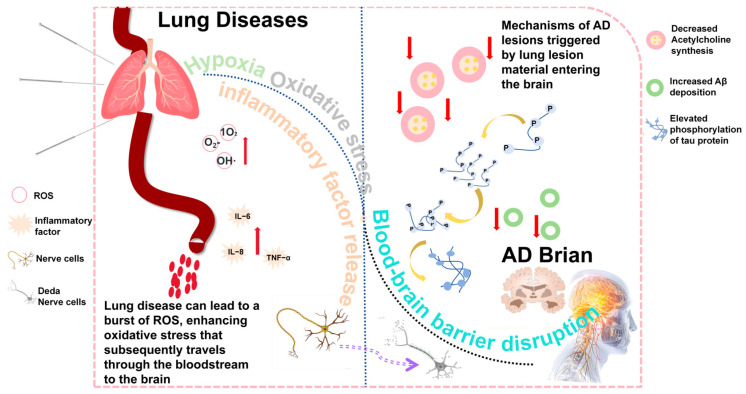
The role of pulmonary diseases in AD. The diagram illustrates the proposed pathway through which pulmonary diseases contribute to the development of AD. In patients with pulmonary conditions such as COPD and OSA, levels of oxidative stress markers (e.g., hydroxyl radical (OH·), singlet oxygen (^1^O_2_), and superoxide anion (O_2_·^−^)) increase in the bloodstream. Concurrently, levels of pro-inflammatory cytokines, including interleukin-6 (IL-6), tumor necrosis factor-alpha (TNF-α), and interleukin-8 (IL-8), are elevated. These factors can trigger several pathological mechanisms, including immune dysregulation, disruption of the blood–brain barrier, and exacerbated oxidative stress, which ultimately lead to neuronal apoptosis, increased amyloid-beta (Aβ) deposition, and protein misfolding, thereby contributing to the onset or progression of AD.

Moreover, COPD patients often present with a chronic systemic inflammatory state. According to research by Fanrong Liang et al., COPD may disrupt the gut microbiota homeostasis, activate the TLR4/NF-κB signaling pathway, and induce inflammation along the gut–lung–brain axis, thereby further exacerbating neuroinflammation [36,37]. This mechanism is also well-documented in AD pathogenesis. Furthermore, long-term smoking and hypoxia lead to elevated levels of reactive oxygen species (ROS), exacerbating oxidative DNA damage (e.g., increased 8-hydroxy-2′-deoxyguanosine) and mitochondrial dysfunction. These changes may encourage amyloid-beta (Aβ) deposition and tau protein hyperphosphorylation [38] (Figure 2).

**Figure 3:**
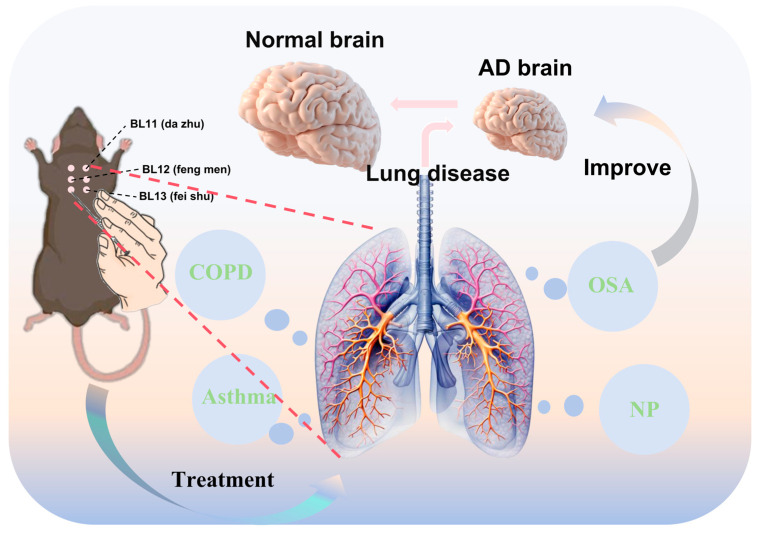
Acupuncture at the six dorsal acupoints may potentially ameliorate AD by modulating pulmonary diseases (COPD: chronic obstructive pulmonary disease; OSA: obstructive sleep apnea; NP: non-pneumonia). The diagram illustrates the location of the six dorsal acupoints on the back, which correspond to the projection zones of the lungs. Stimulating these six acupoints through acupuncture can effectively treat pulmonary diseases. This intervention is hypothesized to exert a beneficial effect on AD following the treatment of pulmonary conditions, thereby potentially promoting the restoration of normal brain function in AD.

In addition to these mechanisms, COPD and AD share dysregulation across multiple molecular pathways, such as aberrant lipid metabolism, hyperactivation of the mTOR pathway, and impaired mitophagy. The lung–brain axis hypothesis is strongly supported by these mechanisms, which collectively show that COPD probably affects AD pathogenesis via multi-target pathways (Figure 3).

### 2.2. Asthma as a Potential Modifiable Risk Factor for Alzheimer’s Disease

Asthma is a chronic inflammatory disease of the airways characterized primarily by airway hyperresponsiveness and reversible airflow limitation. In a study of 202 adult asthma patients who were evaluated using the Montreal Cognitive Assessment (MoCA), Young-Hee Nam et al. found that longer asthma duration is associated with a higher risk of mild cognitive impairment (MCI) [39,40]. Given that MCI is widely regarded as a transitional stage between normal aging and AD, we postulate that asthma may constitute a significant risk factor and exacerbator of Alzheimer’s disease. Notably, Christie M. Bartels et al. found that the incidence and prevalence of AD were significantly higher in asthmatic patients compared with non-asthmatic patients. In addition, a two-year study found that there were 303 additional diagnoses of AD per 100,000 person-years in the asthma cohort. All the aforementioned studies have confirmed that asthmatic patients exhibit a higher susceptibility to AD, which may suggest an underlying mechanistic link between pulmonary pathophysiology and neurodegenerative processes [41].

Pratixa Patel et al. further examined the mechanistic pathway underlying the link between asthma, oxidative stress, and AD. During asthma exacerbations, bronchial epithelial cells and immune cells secrete substantial quantities of ROS [42]. In addition to causing local tissue damage, this also triggers the activation of transcription factors such as NF-κB and AP-1, thereby facilitating the release of pro-inflammatory cytokines including IL-6, IL-8, and TNF-α [43]. These cytokines also play critical roles in AD pathogenesis, accelerating β-amyloid (Aβ) deposition and neuroinflammation activation. Moreover, asthma-associated type 2 immune responses—mediated by cytokines including IL-4, IL-5, and IL-13—have been demonstrated to potentially perturb homeostasis within the central nervous system (CNS) microenvironment. Taken together, these results suggest that asthma may accelerate AD progression through mechanisms involving oxidative stress, inflammatory cytokine release, and immune dysregulation. This supports the lung–brain axis’ pathological function in AD.

### 2.3. Impact of Obstructive Sleep Apnea (OSA) on Alzheimer’s Disease (AD)

The symptoms of obstructive sleep apnea (OSA)—a common breathing disorder related to sleep—include nocturnal intermittent hypoxia, sleep fragmentation, and excessive daytime sleepiness. Experimental studies using AD animal models—notably by Nicola Biagio Mercuri, Claudio Liguori et al.—have shown that the nocturnal induction of intermittent sleep disruption significantly promotes cerebral Aβ deposition [44,45,46]. Additionally, clinical investigations demonstrated a positive correlation between plasma Aβ40 concentrations and the severity of hypoxia in OSA patients [47,48]. Specifically, Aβ40 levels are significantly higher in severe OSA cases compared to those with mild-to-moderate disease. Moreover, pioneering work by David Gozal and colleagues substantiates this pathophysiological nexus. In a rigorously phenotype cohort of 286 children (mean age 7.2 ± 2.7 years), OSA-affected individuals—particularly those with comorbid obesity (OSA + OB)—exhibited significantly elevated circulating concentrations of Aβ42 and presenilin-1 (PS1) (*p* < 0.001 versus age-matched controls). These data demonstrate unequivocally that pediatric OSA dysregulates key Alzheimer’s-associated biomarkers and further indicate that the neurodegenerative cascade is accelerated during prepubertal stages in affected individuals [49].

Multiple longitudinal studies have demonstrated that OSA contributes to the development of pathological features of (AD) through mechanisms like intermittent hypoxia (IH), sleep fragmentation (SF), and excessive daytime sleepiness (EDS). It not only induces the abnormal accumulation of Aβ42 and tau proteins—resulting in neuronal damage—but also triggers hypertension, dysregulated glucose metabolism, chronic inflammation, and oxidative stress [50]. SF, in turn, disrupts sleep architecture and impairs the metabolism and clearance of neurotoxic proteins (e.g., Aβ and phosphorylated tau [P-tau]) in the brain, thereby accelerating neurodegenerative processes. As an indirect measure of the severity of nocturnal respiratory disturbances, EDS presents as diminished attention and reduced information-processing efficiency [51].

Research by Kuo et al. confirmed abnormal alterations in cerebrospinal fluid (CSF) levels of Aβ, T-tau, and P-tau in patients with OSA [52]. Using positron emission tomography (PET), Bubu et al. documented aberrant β-amyloid metabolism in the brains of OSA patients. Concurrently, elevated T-tau and P-tau levels were noted, which increased the neuropathological burden overall [53]. Moreover, OSA significantly alters CSF lactate levels and the Aβ42/T-tau ratio, which suggests that the stability of the cerebral metabolic environment is compromised. Interestingly, OSA patients exhibit a higher carrier rate of AD-risk genes such as apolipoprotein Eε4 (APOE ε4), alongside an elevated body mass index (BMI). These factors collectively increase susceptibility to AD.

Animal studies corroborate this association. In rodent models of OSA, Kim et al. noted hippocampal neuronal degeneration and disorganization, concomitant with significant cognitive decline; additionally, miRNA sequencing demonstrated an elevated expression of miR-132-5p and miR-137-5p—both implicated in AD pathology [54]. Mechanistically, miR-132-5p facilitates cognitive impairment by diminishing acetylcholinesterase (AChE) expression, whereas miR-137-5p intensifies Aβ-induced neurotoxicity.

In summary, OSA adversely affects cerebral health through various mechanisms. It facilitates Aβ accumulation, tau protein phosphorylation, neuroinflammation, and metabolic dysregulation, potentially acting as a significant peripheral contributor to AD pathogenesis.

### 2.4. Mechanistic Impact of Pulmonary Infection and Inflammation on Alzheimer’s Disease (AD)

Pulmonary infections (e.g., bacterial pneumonia) trigger systemic inflammatory responses, producing substantial quantities of pro-inflammatory cytokines such as IL-1β, IL-6, and TNF-α [55]. These cytokines infiltrate the central nervous system (CNS) through the bloodstream, traverse the blood–brain barrier (BBB), activate microglia, and instigate neuroinflammation—ultimately promoting Aβ deposition and tau protein phosphorylation. Notably, Gram-negative bacterial infections can release endotoxins like lipopolysaccharide (LPS), worsening mitochondrial damage and BBB disruption, thereby further compromising neuronal integrity. Employing a Pseudomonas aeruginosa pneumonia mouse model, Kevin R. Nash and Sarah Y. Yuan demonstrated that infected mice manifested pulmonary edema, leukocytosis, and systemic inflammation [56]. These inflammatory mediators disrupt tight junction proteins (e.g., VE-cadherin, claudin-5), significantly enhancing BBB permeability. This facilitates the entry of the inflammatory cells and neurotoxic substances into brain tissue, promoting neuroinflammation and cognitive dysfunction [57].

Research by Anu Chacko et al. further illustrates that Chlamydia pneumoniae infection not only induces pulmonary inflammation but also disseminates into the central nervous system through the olfactory and trigeminal nerves [58]. Within neural cells, it generates inclusions and induces localized Aβ deposition and the abnormal expression of AD-associated genes, while activating β-secretase (BACE) to expedite the amyloid cascade.

Pulmonary infections collectively expedite AD pathogenesis via various mechanisms—including inflammatory mediators, blood–brain barrier disruption, and microbial microinvasion—thereby offering substantial evidence for the lung–brain axis theory [59,60].

### 2.5. Synthesis and Integration of Key Mechanisms

Pulmonary-related disorders are closely associated with AD pathogenesis through multiple pathological mechanisms:

(1) Chronic hypoxia causes structural damage to the hippocampus, severely hindering cognitive function;
(2) Systemic inflammation and immune activation, facilitated by pathways like TLR4/NF-κB and cytokines including interleukin-6 (IL-6) and tumor necrosis factor-alpha (TNF-α), propel neuroinflammation;
(3) Oxidative stress and mitochondrial dysfunction, indicated by increased reactive oxygen species (ROS) and 8-hydroxy-2′-deoxyguanosine (8-OHdG), facilitate Aβ and tau pathologies;
(4) Metabolic anomalies and signaling irregularities—illustrated by lipid dysmetabolism, mTOR activation, and ceramide accumulation—contribute to AD pathogenesis;
(5) Emerging mechanisms such as the imbalance in miRNA expression regulation are also worthy of attention.

**Figure 4:**
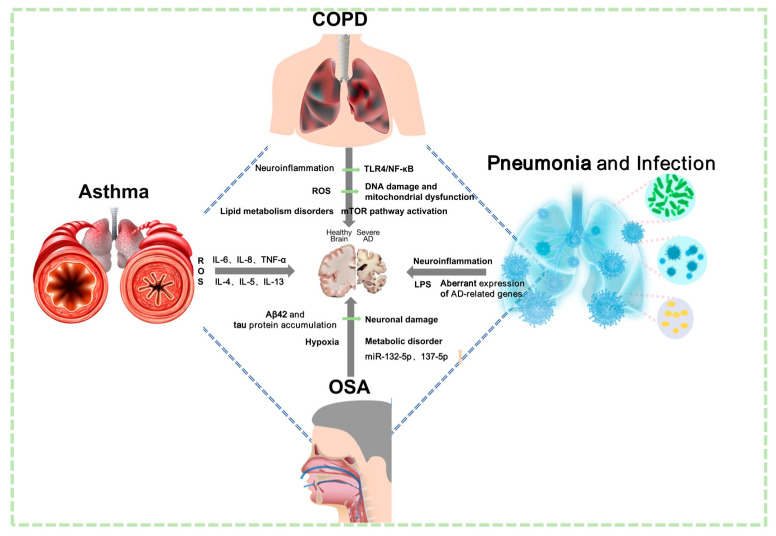
Potential mechanisms linking common pulmonary diseases to AD. COPD may contribute to cerebral pathology through multiple mechanisms, including increased ROS, neuroinflammation, lipid metabolism disorders, mTOR pathway activation, DNA damage, mitochondrial dysfunction, and activation of the TLR4/NF-κB pathway. Asthma potentially impacts the brain via significant oxidative stress and the release of inflammatory mediators such as IL-6, IL-8, TNF-α, IL-4, IL-5, and IL-13. OSA could influence brain function through mechanisms such as accumulation of Aβ42 and tau protein, chronic hypoxia, neuronal damage, metabolic disorders, and dysregulation of miRNAs including miR-132-5p and miR-137-5p. Infectious pulmonary diseases (e.g., non-pneumonia, NP) may induce or exacerbate AD via pathways involving neuroinflammation, LPS-mediated effects, and aberrant expression of AD-related genes.

In summary, current substantial evidence indicates that pulmonary pathologies directly and/or indirectly contribute to Alzheimer’s disease pathogenesis and progression. Consequently, incorporating pulmonary considerations into AD therapeutic strategies is imperative. However, the existing literature demonstrates a critical gap in robust clinical evidence supporting pulmonary-focused interventions for AD amelioration. This evidentiary void precisely constitutes the conceptual innovation of our study—establishing a novel therapeutic paradigm through acupuncture-mediated pulmonary modulation to mitigate Alzheimer’s pathology. The “lung–brain axis” provides a conceptual framework for multi-organ crosstalk. The axis provides innovative insights into the pathogenesis of AD and identifies potential targets with scientific rationale for TCM and acupuncture-based AD interventions (Figure 4).

## 3. The Potential of Acupuncture as a Modulator of the Lung–Brain Axis: Mechanistic Hypotheses and Translational Challenges

Acupuncture, a significant component of TCM, has garnered extensive experience in managing pulmonary disorders and enhancing cognitive function [61]. Contemporary research indicates that acupuncture at designated points enhances pulmonary function and reduces inflammation, while also influencing cerebral activity via the neuro–immune–endocrine pathway, with the potential to regulate the “lung–brain axis”. Xiao Zhao Li et al. discovered that acupuncture in the thoracic vertebral region (C7-T5), where the dorsal root ganglion is situated, markedly enhanced pulmonary ventilation in asthma patients; furthermore, the relevant acupoints, such as the loom, the wind gate, and the lung point, corresponded highly to the traditional “dorsal acupoints” of TCM. Furthermore, a study by Wei Zhang et al. demonstrated that acupuncture at Baihui (GV20) could down-regulate GSK-3β expression and up-regulate GAP-43 levels in the hippocampus of rats, thereby enhancing cognitive function and facilitating synaptic repair [62]. This finding hints at possible mechanisms through which acupuncture might simultaneously target pathways relevant to both pulmonary and neurological diseases.

As summarized by Peter Jenner et al., acupuncture treatment in Alzheimer’s disease patients significantly enhances activity in the temporal and prefrontal lobes—regions associated with memory and cognitive function [62]. In addition, a study on an ovalbumin (OVA)-induced asthma mouse model confirmed that acupuncture can effectively improve airway hyperresponsiveness in mice, reduce lymphocyte counts in bronchoalveolar lavage fluid (BALF), and alleviate airway inflammation [63]. Collectively, these findings indicate that acupuncture possesses the ability to restore immune homeostasis and inhibit inflammation. Nonetheless, there remains insufficient research on the mechanism of acupuncture grounded in the theory of “lung–brain axis”.

The six dorsal acupoints in the Lingnan school of acupuncture, “Jin Sanzhen”, specifically the loom (BL11), the wind gate (BL12), and the lung (BL13), are extensively utilized in the management of lung diseases, and are anticipated to have a significant potential for intervention through the modulation of the lung–brain axis. While the “Jin Sanzhen” needling technique is widely employed in managing lung diseases, its underlying mechanisms—particularly those related to the lung–brain axis—remain to be fully elucidated. Current evidence directly validating this pathway is limited due to constraints in sample sizes, clinical study design, and technical challenges, highlighting a critical area for future investigation.

### 3.1. Study on the Modern Mechanism of Six Dorsal Acupoints Interfering with Lung Function

The anatomical site of the region where the “dorsal six points” are located significantly coincides with the dorsal root ganglion (C7-T5) [64]. The dorsal root ganglion (DRG) plays a role in pulmonary function. In a study by David Richard Springall et al., the researchers used a combination of retrograde axonal tracing and immunohistochemistry. True Blue was injected into the extrapulmonary airways and administered percutaneously into the left and right lungs. Retrogradely labeled perikaryal were detected in the DRG, and CGRP-immunoreactive cells were found exclusively in the vagus nerve and DRG. These results confirm a strong connection between the DRG and the lungs. Furthermore, the six dorsal points (BL11, BL12, BL13) show a precise anatomical correspondence with the ganglion, supporting the existence of a retrograde regulatory pathway between acupuncture points and the lungs [65]. Contemporary studies have demonstrated that pulmonary disorders like asthma can stimulate airway epithelial cells to produce neurotrophic factors (e.g., NGF), which activate DRG via retrograde axonal transport, enhance the production of inflammatory neuropeptides such as substance P (SP), induce neurogenic inflammation, and exacerbate the pathological changes in the lung. Acupuncture of the Lung Yu point can diminish the absorption of NGF by DRG via mechanical stimulation, decrease SP synthesis, and alleviate airway inflammation and smooth muscle spasms [66].

Furthermore, acupuncture at the six dorsal points can modulate vagal tone, enhance acetylcholine release, and suppress inflammatory cell activity. Wang et al. discovered that acupuncture at the lung acupoints markedly suppressed the expression of Th2-type cytokines (IL-4, IL-5, and IL-13) and epithelial-derived factors (IL-25, IL-33, and TSLP) in a rat model, diminished airway inflammatory cell infiltration, and improved lung tissue architecture [67].

**Figure 5:**
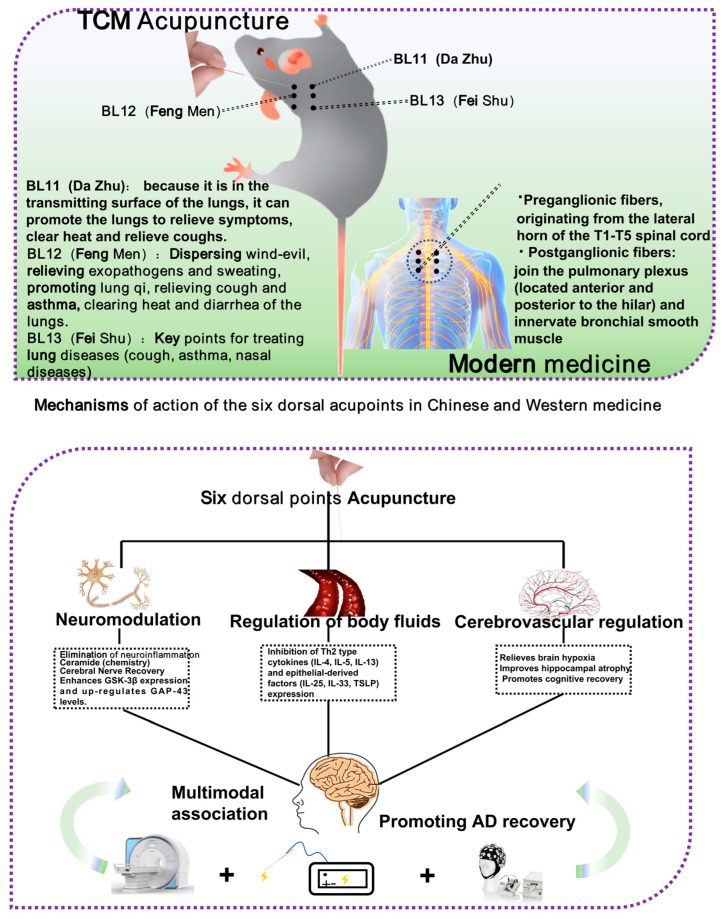
Research on acupuncture at the “BL11, 12, 13” for treating AD via the “lung–brain axis” has evolved from initial empirical observations to ongoing mechanistic investigations. Future studies should incorporate advanced technologies—such as functional magnetic resonance imaging (fMRI), electroacupuncture, and wearable EEG devices—to further elucidate the underlying mechanisms and optimize acupuncture-based therapeutic strategies targeting this axis.

The Fengmen (BL12) acupoint participates in the modulation of inflammatory signaling pathways; acupuncture at this site can down-regulate the expression of CCL1, CCL8, and TSLP mRNA, diminish excessive airway remodeling, and inhibit the expression of α-SMA in fibroblasts [68]. Numerous animal studies have demonstrated that acupuncture at the six dorsal acupoints exhibits considerable anti-inflammatory effects, antioxidative stress activity, and enhancements in ventilatory function, and its mechanism of action is highly compatible with the pathways of oxidative stress and neuroinflammation involved in the pathogenesis of AD (Figure 5).

### 3.2. Potential Role of the Six Dorsal Acupoints in Modulating Lung Disease–AD Cross-Pathology

The six dorsal acupoints not only enhance local lung function but also systematically modulate oxidative stress and inflammatory signaling pathways. Wei Gao et al. discovered that the expression of SP, NKA, and NKB diminished, whereas the level of vasoactive intestinal peptide (VIP) increased in the lung tissue of rats with an asthma model after acupuncture at BL11, BL12, and BL13, indicating that it may relax smooth muscle and suppress inflammation through the up-regulation of the cAMP signaling axis. In Chinese medicine theory, BL13 is an essential component of smooth muscle and suppresses inflammatory responses [69]. Ida Nurwati et al. also provided evidence supporting this perspective by demonstrating that acupuncture at BL13 ameliorated airway remodeling in a mouse model of chronic asthma. After two weeks of treatment, acupuncture significantly reduced the thickness of the bronchial epithelium and smooth muscle, and decreased the number of goblet cells in the bronchioles [70].

In Chinese medical theory, BL13 is the dorsal yu of the lung, responsible for regulating lung qi and enhancing positive qi; BL12 dispels wind, relieves epidemics, and promotes lung qi; BL11 is classified as the “Bone Hui” of the eight points, facilitating the repair of lung tissues through the regulation of qi and blood. Contemporary studies have demonstrated that the injection of autologous blood into the above points can stimulate the release of immunoglobulin, antagonize histamine, acetylcholine, and other mediators, and mitigate oxidative damage to the lungs.

Moreover, acupuncture at the six dorsal points can enhance pulmonary ventilation. Tsai et al. discovered a significant correlation between respiratory impairment indicators in OSA patients and the risk of AD [71]. Li and Fei indicated that PaO_2_ and SaO_2_ were highly correlated with hippocampal volume and S100B levels in COPD patients [33,72]. Lai et al. demonstrated that acupuncture at the Lung Yu (BL13) combined with the Tianshu (ST25) could markedly enhance PEF, FEV1/FVC, and other indexes, improving hypoxia and indirectly mitigating AD risk factors [73].

### 3.3. The Potential Impact of Stimulating Additional Acupoints Along the Lung Meridian on Cerebral Functions

The Back-Shu points (BL11, BL12, and BL13), as the primary corresponding points of the lung on the back, are considered to exert a direct therapeutic influence on the lung organ. Consequently, they have been designated as the key focus of this investigation. However, according to Traditional Chinese Medicine theory, two Lung Meridians also traverse the anterior aspect of the upper limbs. The acupoints located along these meridians represent equally critical avenues for research.

As investigated by the research team of Xianghua MDb, 36 volunteers with severe anxiety disorders were divided into three groups, one of which received acupuncture at the “Yuji” (LU10) acupoint. Magnetic resonance imaging (MRI) was then employed to monitor brain responses during the acupuncture stimulation. “Yuji” (LU10), a significant distal point of the Lung Meridian located on the hand, is traditionally indicated in Traditional Chinese Medicine (TCM) for improving respiratory function, alleviating cough, and regulating mood for emotional relaxation. Comparative analysis with the control group revealed that acupuncture at LU10 activated several brain regions, including the left frontal lobe, medial frontal gyrus, temporal lobe, and hippocampus. The researchers hypothesize that this intervention may modulate anxiety by up-regulating or down-regulating activity within specific relevant brain regions or networks [74]. Furthermore, Rong-lin Cai, Ling Hu, and colleagues have also observed that acupuncture applied to the Lung Meridian may exert certain effects on the brain. The research team administered electroacupuncture stimulation at the “Taiyuan” (LU9) acupoint in rats and found that treated rats exhibited increased dopamine secretion in the hypothalamus. Substantial existing research has demonstrated that dopamine is implicated in Alzheimer’s disease and other central nervous system disorders. The ability of acupuncture at LU9 to modulate dopamine release may potentially serve as a complementary mechanism within the proposed “acupuncture–lung–brain axis” regulatory pathway [75]. Additionally, acupuncture may potentially modulate depression through the “lung–brain axis.” In a study conducted by Yi Wang, Dilinuer Abulikemu, and colleagues, 160 patients with mild to moderate depression were randomly assigned to either a medication-only group or a combined acupuncture and medication group. The acupuncture group received stimulation at “Shaoshang” (LU11), an acupoint belonging to the Lung Meridian that is traditionally utilized in the treatment of mental health disorders and is functionally associated with the lung organ. The results demonstrated that the combined treatment group achieved an efficacy rate of 97.5%, significantly higher than that of the medication-only group. Furthermore, more pronounced elevations in neurotransmitter levels—specifically serotonin (5-HT), dopamine (DA), and norepinephrine (NE)—were observed in the combination group. These findings suggest that acupuncture at specific acupoints of the Lung Meridian can induce measurable changes in brain neurochemistry, supporting its potential role in modulating depressive pathology [76].

This section postulates the potential existence of an “acupuncture–lung–brain axis” by reviewing evidence that acupuncture at acupoints on the Lung Meridian can induce cerebral changes. Although accumulating evidence supports this proposition, several issues remain unresolved. These include the unclear mechanisms underlying acupuncture’s effects on the nervous system, visceral organs, and physiological responses, as well as the general lack of foundational experimental validation within Chinese medicine theory. Further empirical support is therefore necessary. Identifying direct therapeutic mechanisms or neural pathways constituting the “lung–brain axis” would provide a more robust scientific foundation for the efficacy of acupuncture.

### 3.4. The “Lung–Brain Axis” Mechanism of Action Has Been Shown to Be a Viable Therapeutic Strategy

Previous sections have demonstrated that acupuncture at the Back-Shu points (BL11, BL12, BL13) can improve pulmonary function and reduce levels of factors implicated in AD pathogenesis, such as substance P (SP). However, robust evidence supporting a “lung–brain axis” mechanism remains limited. This section therefore reviews existing findings related to the lung–brain axis to substantiate the hypothesis that acupuncture at these points alleviates Alzheimer’s disease via this pathway. Professor Alexander Flügel, Francesca Odoardi, and colleagues used neomycin to shift the lung microbiota toward a dominance of LPS-rich bacterial phyla. They observed that microglia in the brain adopted a type I interferon signaling-associated gene expression profile, which significantly suppressed systemic inflammation—a key contributor to Alzheimer’s disease pathogenesis [77]. This aligns with the aforementioned findings in this study that acupuncture at the Back-Shu points reduces systemic inflammation in patients with infectious pneumonia by modulating neurotransmitters and inflammatory responses. Andy Peng Xiang et al. established a mouse model of depression and administered mesenchymal stromal cell-derived brain-derived neurotrophic factor (BDNF) to stimulate pulmonary sensory neurons. They found that the activation of tropomyosin receptor kinase B (TrkB) alleviated depressive behaviors, revealing a mechanistic pathway, “pulmonary vagal afferent nerves → nucleus tractus solitarius → dorsal raphe nucleus”, that mediates antidepressant and anxiolytic effects. This finding further supports the feasibility of targeting the lung–brain axis for Alzheimer’s disease treatment [78].

### 3.5. The Influence of Acupuncture at Lung-Related Acupoints on Cerebral Function Is Proposed to Operate Through Specific, Though Not Yet Fully Elucidated, Underlying Mechanisms

In the preceding sections, multiple lines of evidence have been presented supporting the role of acupuncture in regulating brain disorders via the “lung–brain axis”; however, the integration of these underlying mechanisms has yet to be systematically discussed. Primarily, the modulation of brain function through acupuncture at lung-related acupoints is likely intricately associated with neural mechanisms. In a study by Minhao Zhang, electroacupuncture was applied to the dorsal root ganglion (DRG) in a mouse model of neuropathic pain. Neural injury significantly up-regulated autophagy levels in DRG macrophages, whereas acupuncture treatment attenuated neuropathic pain (NP), potentially by promoting AMPK/mTOR-mediated autophagy within these macrophages, thereby alleviating pain. Within pain research, the DRG plays a crucial role in the conduction of peripheral primary afferent neurons. Importantly, as previously demonstrated, there is a notable anatomic correspondence between the locations of the Back-Shu points (BL11, BL12, and BL13) and the DRG. It is therefore reasonable to postulate that acupuncture at these Back-Shu points may exert therapeutic effects on the brain through neuromodulatory mechanisms [79].

Secondly, acupuncture may exert its therapeutic effects via the “lung–brain axis” by modulating neuroendocrine activity. As noted by Zhihui Hao et al., acupuncture at BL-13 in rat models of allergic asthma was associated with the inhibition of acetylcholine synthesis and release. Concurrently, acupuncture influences monoamine neurotransmitters; for instance, stimulation at DU-16 increased levels of serotonin (5-HT), norepinephrine (NA), and dopamine (DA) in brain tissue, thereby ameliorating symptoms in AD model mice. Therefore, the mechanisms through which acupuncture at lung-related acupoints influences cerebral function may also be interpreted from a neuroendocrine perspective, warranting further investigation [80].

Finally, the role of acupuncture in the “lung–brain axis” may also be mediated through the modulation of systemic inflammation. In research by Zhongxi Lyu et al., acupuncture was shown to exert anti-inflammatory effects by promoting the polarization of macrophages from the M1 to the M2 phenotype, regulating the balance of T cell populations and enhancing superoxide dismutase (SOD) activity via the Nrf2/HO-1 pathway to suppress oxidative stress and scavenge oxygen free radicals. Additionally, following acupuncture, the remodeling of connective tissue was accompanied by the up-regulated secretion of various molecules, which in turn activates NF-κB, MAPK, and ERK pathways in mast cells, monocytes, and macrophages, thereby contributing to anti-inflammatory responses [66]. There is established evidence linking pulmonary inflammation to the onset and progression of AD. Conditions such as COPD, OSA, and asthma are associated with the release of pro-inflammatory cytokines that can traverse the blood–brain barrier and contribute to neural damage. Therefore, we hypothesize that acupuncture at lung-related acupoints may also activate anti-inflammatory pathways, leading to systemic immunomodulation that could confer therapeutic benefits for treating or alleviating AD.

### 3.6. Current Limitations and Methodological Challenges

While the hypotheses and preliminary evidence for acupuncture’s modulation of the AD-related lung–brain axis have been systematically reviewed and show a certain foundation, it is crucial to acknowledge the significant limitations and methodological challenges that remain in this field to guide future research.

(1) Predominance of preclinical evidence and mechanistic speculation: Currently, the majority of evidence supporting the “acupuncture–lung–brain axis” concept is derived from animal studies. Although these models are invaluable for exploring initial mechanisms (e.g., neuroinflammation, oxidative stress), their translational relevance to the complex pathophysiology of human AD remains unproven. Many proposed mechanisms, such as the precise neuromodulatory pathways from the dorsal root ganglion to the hippocampus, remain speculative and lack direct validation in human subjects.
(2) Lack of standardized protocols and high-quality clinical trials: Clinical research in this area is characterized by a critical shortage of large-scale, rigorous randomized controlled trials (RCTs). The existing human studies often suffer from small sample sizes, insufficient blinding (practitioner and patient), and a lack of appropriate sham–acupuncture controls that can adequately account for placebo effects. Furthermore, there is a notable absence of standardized acupuncture treatment protocols regarding needle manipulation techniques, session frequency and duration, and acupoint selection criteria (e.g., using Back-Shu points alone versus combining them with limb points). This heterogeneity makes it difficult to compare results across studies and draw definitive conclusions.
(3) Insufficient integration with modern biomedical frameworks: Although anatomical correspondences (e.g., between Back-Shu points and the dorsal root ganglion) are highlighted, the field often struggles to move beyond correlation to establish causation. The molecular and neural pathways linking acupuncture stimulation at a specific point to a systemic anti-inflammatory effect or a change in cerebral function are not yet fully mapped. There is a pressing need to integrate traditional concepts like “regulating lung qi” with testable modern biological measures, such as specific changes in vagal nerve activity, cytokine profiles, or neuroimaging biomarkers, to build a more robust and universally comprehensible scientific foundation.

In summary, while the concept of using acupuncture to modulate the lung–brain axis for AD treatment is theoretically compelling and supported by fragmentary evidence, the field is currently in its infancy. Overcoming these limitations will require a concerted effort towards designing methodologically sound clinical trials, employing advanced neuroimaging and molecular techniques to elucidate mechanisms, and fostering a deeper integration of traditional knowledge with contemporary neuroimmunology. Addressing these challenges is paramount for transforming this promising hypothesis into a validated therapeutic strategy.

## 4. Evidence Summary and Future Directions

Numerous studies have been undertaken on the direct treatment of Alzheimer’s disease and pulmonary diseases through acupuncture, with a thorough examination of the mechanisms and pathways involved. Nevertheless, limited research has been undertaken on the “lung–brain” axis through acupuncture, both domestically and internationally, necessitating extensive investigations to address the deficiencies in this area. Traditional Chinese Medicine prioritizes the investigation of disease origins from a holistic viewpoint, suggesting that the “lung–brain” axis may offer a novel framework for comprehending the pathogenesis of Alzheimer’s disease. Nonetheless, current research typically suffers from inadequate sample sizes, unidentified mechanisms, and a lack of multi-center clinical validation, which limit the wide application of acupuncture to regulate the lung–brain axis in the prevention and treatment of AD.

Future research may concentrate on cross-system and multimodal evidence integration, particularly investigating the associative mechanisms between OSA and AD in both epidemiological and clinical contexts. For instance, Tsai et al. discovered in a prospective study that AHI, ODI-3%, and R-ArI were markedly elevated in patients of the OSA high-risk group than those in the low-risk group, and that for every standard deviation increase in AHI, AD markers Aβ42 × T-Tau increased 1.13-fold, suggesting that the “intermittent hypoxia-arousal response” may exacerbate AD pathology. Lutsey et al. demonstrated that patients with COPD exhibited an elevated risk of developing AD, and that hippocampal volume was markedly diminished on MRI [81].

The “six dorsal acupoints + fMRI” methodology, in conjunction with an electrical stimulation research approach, can facilitate systematic experiments for mechanism validation. The study design may encompass 40 pulmonary patients with mild MCI and 20 healthy controls, who will be randomly assigned to receive either electrical stimulation or sham stimulation at authentic acupoints. This will be complemented by resting-state and task-state fMRI to document alterations in brain function and evaluate the correlations among pulmonary function (FEV1, PEF), cognitive function (MMSE, MoCA), and serum biomarkers (Aβ42, p-Tau).

Imaging analysis can assess the activation intensity of lung-associated brain regions including the insula, pontine respiratory center, hippocampus, and dorsolateral prefrontal cortex (DLPFC), calculate lung–brain functional connectivity (e.g., correlation r-value between insula and hippocampus), and analyze the changes in cerebral perfusion levels using perfusion imaging (PWI), so as to elucidate the relationship between the dynamics of cerebral blood flow and the effects of needling.

Regarding the mechanism dimension, three intersecting pathways may also be emphasized: (1) Neuromodulation pathway: Acupuncture at the six dorsal acupoints stimulated the spinal afferent fibers of the T3-T5 segment, subsequently modulating the nucleus tractus solitarius and the reticular formation and enhancing the excitability of neurons in the hippocampal region. Imaging evidence demonstrated a positive correlation between brainstem and hippocampal BOLD signals during acupuncture. (2) Humoral–inflammatory regulation: Acupuncture markedly decreased serum IL-6 levels and enhanced the expression of Aβ clearance receptor LRP1 in the brains of COPD patients. The reduction in IL-6 exhibited a negative correlation with the degree of prefrontal lobe activation. (3) Enhanced cerebral hemodynamics: Enhanced pulmonary function resulted in increased cerebral perfusion, and the BOLD signal exhibited a positive correlation with the MMSE score.

In conclusion, in the future, we should systematically incorporate multimodal indicators, including epidemiology, fMRI, cognitive assessments, and inflammatory markers, alongside contemporary technology to investigate the causal relationship among acupuncture, the lung–brain axis, and AD. This approach aims to enhance the modernization of the clinical applications of traditional acupuncture and to provide innovative means and theoretical foundations for the intervention of Alzheimer’s disease. We will offer innovative methods and theoretical foundation for the intervention of Alzheimer’s disease.

## 5. Conclusions

With the accelerating global aging process, the incidence of AD continues to rise, presenting a major public health challenge worldwide. While traditional research has predominantly focused on mechanisms within the central nervous system (CNS), recent studies indicate that the progression of AD is closely associated with various systemic diseases—particularly pulmonary disorders. These respiratory conditions contribute to the pathological process of AD through multiple pathways such as hypoxia, inflammation, and oxidative stress, thereby highlighting the potential significance of the “lung–brain axis” in the pathogenesis of AD.

Building upon this perspective, this study systematically reviews epidemiological and pathological evidence linking common pulmonary diseases—including COPD, asthma, sleep apnea syndrome, and pulmonary infections and inflammation—to AD. It aims to elucidate how impaired lung function may influence cognitive function and neurodegenerative processes through diverse biological mechanisms.

Subsequently, this study reviews the therapeutic potential of acupuncture in ameliorating common pulmonary disorders. It further examines how stimulation at specific acupoints—such as BL11, BL12, and BL13, which correspond to dorsal projection zones of the lung, as well as along the Lung Meridian traversing the anterior aspect of the upper limbs—can modulate and influence cerebral functions. These analyses aim to strengthen the scientific basis for acupuncture’s role in treating pulmonary and neurological conditions, thereby providing a theoretical foundation for the “lung–brain axis” hypothesis. However, limitations such as sample size and ongoing debates surrounding acupuncture methodology indicate several areas requiring improvement in the current research.

In future investigations, our research group will focus on identifying the specific pathways related to AD that are either activated or suppressed during acupuncture stimulation at lung-associated acupoints, moving beyond merely observing changes in biomarkers and metabolites. Follow-up studies should strive to integrate mechanisms underlying the cross-system intervention chain of “acupuncture–lung–brain axis–AD.” This will entail incorporating advanced methodologies such as MRI, serum biomarkers, neuroimaging, cognitive assessments, and pulmonary function metrics, thereby establishing a multi-modal, cross-scale research framework.

In conclusion, acupuncture’s potential in treating AD via the lung–brain axis represents a highly significant research direction. Subsequent studies should employ large-scale, multi-center randomized controlled trials to validate the clinical efficacy and specific mechanisms of acupuncture intervention, facilitating its transition from an adjunct therapy to a core treatment strategy. This approach not only broadens the theoretical paradigm for AD treatment but also provides novel insights into the contemporary application of traditional acupuncture in the management of neurodegenerative disorders.

## Data Availability

Data are contained within the article. The data presented in this study can be requested from the authors.
